# Oxidation of Molecular Hydrogen by a Chemolithoautotrophic Beggiatoa Strain

**DOI:** 10.1128/AEM.03818-15

**Published:** 2016-04-04

**Authors:** Anne-Christin Kreutzmann, Heide N. Schulz-Vogt

**Affiliations:** a Max Planck Institute for Marine Microbiology, Bremen, Germany; b Leibniz Institute for Baltic Sea Research Warnemuende (IOW), Rostock, Germany; University of Tennessee and Oak Ridge National Laboratory

## Abstract

A chemolithoautotrophic strain of the family Beggiatoaceae, Beggiatoa sp. strain 35Flor, was found to oxidize molecular hydrogen when grown in a medium with diffusional gradients of oxygen, sulfide, and hydrogen. Microsensor profiles and rate measurements suggested that the strain oxidized hydrogen aerobically when oxygen was available, while hydrogen consumption under anoxic conditions was presumably driven by sulfur respiration. Beggiatoa sp. 35Flor reached significantly higher biomass in hydrogen-supplemented oxygen-sulfide gradient media, but hydrogen did not support growth of the strain in the absence of reduced sulfur compounds. Nevertheless, hydrogen oxidation can provide Beggiatoa sp. 35Flor with energy for maintenance and assimilatory purposes and may support the disposal of internally stored sulfur to prevent physical damage resulting from excessive sulfur accumulation. Our knowledge about the exposure of natural populations of Beggiatoaceae to hydrogen is very limited, but significant amounts of hydrogen could be provided by nitrogen fixation, fermentation, and geochemical processes in several of their typical habitats such as photosynthetic microbial mats and submarine sites of hydrothermal fluid flow.

**IMPORTANCE** Reduced sulfur compounds are certainly the main electron donors for chemolithoautotrophic Beggiatoaceae, but the traditional focus on this topic has left other possible inorganic electron donors largely unexplored. In this paper, we provide evidence that hydrogen oxidation has the potential to strengthen the ecophysiological plasticity of Beggiatoaceae in several ways. Moreover, we show that hydrogen oxidation by members of this family can significantly influence biogeochemical gradients and therefore should be considered in environmental studies.

## INTRODUCTION

Members of the family Beggiatoaceae are colorless sulfur bacteria known to oxidize reduced sulfur compounds and organic substances for chemolithoautotrophic, chemoorganoheterotrophic, and mixotrophic growth (1). The use of various organic substances, such as mono- and dicarboxylic acids, sugars, amino acids, and alcohols, has been studied repeatedly in different strains of the family (2–6), but inorganic electron donors other than reduced sulfur compounds were never reported to support growth. The only indication of the oxidation of a nonsulfuric, inorganic electron donor was the stimulation of sulfur reduction by molecular hydrogen in a microaerophilic Beggiatoa strain under short-term anoxic conditions (7). Hydrogen oxidation or hydrogen-supported growth has been reported for many other well-known sulfur oxidizers such as members of the families Chromatiaceae (8), Acidithiobacillaceae (9, 10), Aquificaceae (11–13), and Sulfolobaceae (14), the genus Sulfurimonas (15, 16), the SUP05 clade (17), and endosymbionts of mussels (18). This suggests that hydrogen oxidation may be a widespread metabolic trait among sulfur oxidizers and as such may also be realized in the family Beggiatoaceae.

Substantial amounts of molecular hydrogen are produced and consumed in many microbial habitats, so H_2_ is considered to be an important electron transfer agent in oxic and anoxic environments (19). Nevertheless, there is little information about the environmental exposure of Beggiatoaceae populations to hydrogen and the potential importance of hydrogen oxidation for members of the family *in situ*. Despite high conversion rates, *in situ* studies on hydrogen cycling and availability are difficult due to the generally very low ambient concentrations (20). Steep biogeochemical gradients, which are typical for habitats of Beggiatoaceae, pose an additional problem because these necessitate a sampling resolution on the micrometer scale for meaningful conclusions. Microsensors are typically used for this purpose, and a microsensor for hydrogen has been available for more than two decades (21). However, the hydrogen microsensor has the critical disadvantage of being sensitive to hydrogen sulfide (22). This cross-reactivity disqualifies the sensor from many *in situ* applications, in particular, from measurements in habitats of sulfur bacteria, where the concentrations of sulfide are usually considerably higher than those of hydrogen.

In the present study, we investigated the consumption of molecular hydrogen in cultures of a chemolithoautotrophic Beggiatoa strain using microsensors. Culture-based experiments allowed us to adjust the concentrations of hydrogen and sulfide to levels at which reliable measurements with the hydrogen microsensor are possible. We discuss here how hydrogen oxidation can contribute to the ecophysiological plasticity of the strain and point out environmental settings in which members of the family Beggiatoaceae may be able to use hydrogen as an electron donor and energy source.

## MATERIALS AND METHODS

### Organisms and cultivation.

All experiments were conducted with the marine chemolithoautotrophic bacterium Beggiatoa sp. strain 35Flor, which was maintained in a defined coculture with Pseudovibrio sp. strain FO-BEG1, a heterotrophic and metabolically versatile bacterium (23). The coculture was grown in a medium with opposed gradients of oxygen and sulfide as described previously (24, 25). The concentration of NiCl_2_ in top agar and bottom agar was increased to 7 μM to provide a sufficient amount of nickel for the synthesis of the [NiFe]-hydrogenase cofactor. Bottom agar sulfide concentrations were adjusted to 6 mM (low sulfide flux) or 16 mM (high sulfide flux), depending on the experiment. Ammonium chloride in a concentration of 200 μM was added to the top agar only when the influence of a fixed nitrogen source on hydrogen oxidation was to be tested; nitrate was never added to the medium.

**FIG 1:**
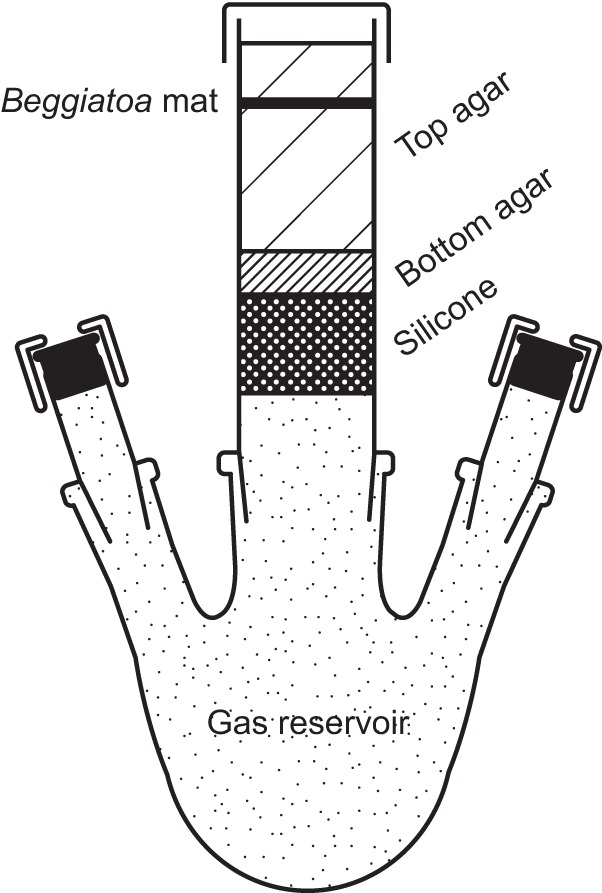
Setup for the incubation of Beggiatoa sp. 35Flor in the presence of diffusional gas gradients. The Beggiatoa mat grows between opposed diffusional gradients of oxygen, sulfide, and (if the latter is provided) hydrogen. Oxygen is supplied to the mat by diffusion from the headspace above, and sulfide is provided by diffusion from the bottom agar below. Hydrogen can be added to the gas reservoir and can reach the mat by diffusion through the silicone plug.

The setup for cultivation in the presence of a diffusional hydrogen gradient was as follows (Fig. 1). A glass tube with a conical ground cone (nominal size [NS] 29/32; 26 by 130 mm; inner diameter 22 mm; all glassware was obtained from Lenz Laborglas GmbH & Co. KG, Wertheim, Germany) was closed toward the cone with a 20-mm-high plug of silicone (RTV-2 silicone, 13 ShA; Silikonfabrik.de, Ahrensburg, Germany) and loosely capped on top with a lid of thick aluminum foil. The sterilized tube was placed on the central socket of a 100-ml three-neck flask. The screw thread adapters on the side necks (NS 14/23) of the flask were closed with butyl stoppers and apertured caps. All joints were greased with medium-viscosity Baysilone paste (GE Bayer Silicones GmbH & Co. KG, Leverkusen, Germany) and fixed in place with steel clips. Bottom agar (4-ml) and top agar (17-ml) layers were poured consecutively onto the silicone plug. The gas reservoir was flushed with either nitrogen or hydrogen gas for 30 min immediately after the pouring of the top agar. In cultures with a high sulfide flux, a lower hydrogen partial pressure was achieved by replacing 12 ml of the nitrogen-filled gas reservoir with hydrogen. Gradients were allowed to establish for 1 day prior to inoculation with 300 μl Beggiatoa filament suspension prepared from mats of 9-to-16-day-old precultures (25). Hydrogen-supplemented and -unsupplemented cultures were prepared in parallel and inoculated with the very same homogeneous filament suspension. The cultures were incubated at room temperature, and the gas reservoirs were refreshed every 3 to 4 days.

### Microsensor measurements.

Microsensors for pH (pH-10), H_2_ (H2-10), O_2_ (OX-10), and H_2_S (H2S-10) with tip diameters of 8 to 12 μm and response times of <10 s were purchased from Unisense A/S (Aarhus, Denmark) and calibrated directly before and after the measurements as described by Schwedt et al. (25). The hydrogen sensor was calibrated in artificial seawater (25) by stepwise addition of a hydrogen-saturated stock solution, the concentration of which was calculated as described by Gordon et al. (26). Profiles of total sulfide (S_tot_ = H_2_S + HS^−^ + S^2−^) were calculated from the corresponding H_2_S and pH profiles as described previously (25, 27). Measured hydrogen profiles were corrected for the H_2_S-derived background recorded by the cross-reactive hydrogen sensor. This background was estimated from profiles measured in hydrogen-unsupplemented parallel cultures. In the case of cultures with a low sulfide flux, the average background for a given H_2_S concentration was calculated from the H_2_S and apparent H_2_ concentrations measured in the very same hydrogen-unsupplemented cultures at the very same depths. The H_2_S-derived background in hydrogen-supplemented cultures was then calculated based on the measured H_2_S profiles and was subtracted from the measured H_2_ profiles. The estimated H_2_S-derived background accounted for ≤12% of the recorded hydrogen signal in all hydrogen-supplemented cultures and was ≤5% in most cases. In cultures with a high sulfide flux, the average H_2_S-derived background profile measured in hydrogen-unsupplemented cultures was directly subtracted from the H_2_ profiles measured in hydrogen-supplemented cultures. This was possible because the oxygen-sulfide interfaces were located at similar depths and H_2_S profiles were essentially congruent in hydrogen-supplemented and -unsupplemented cultures with a high sulfide flux. It has to be noted that both corrections could overestimate the contribution of the H_2_S-induced background, because genuine H_2_ signals present in hydrogen-unsupplemented cultures would wrongly be ascribed to H_2_S and subtracted. It is indeed possible that hydrogen-unsupplemented cultures contained H_2_, because Beggiatoa sp. 35Flor fixes nitrogen under standard cultivation conditions (A.-T. Henze, unpublished data) and this process is associated with the evolution of H_2_ (reviewed in reference 28). However, we assume that the hydrogen concentrations were not significant in hydrogen-unsupplemented cultures due to the slow growth and high hydrogen oxidation rates of Beggiatoa sp. 35Flor. Correspondingly, there was no notable difference in the H_2_ profiles measured in hydrogen-supplemented nitrogen-fixing and non-nitrogen-fixing Beggiatoa sp. 35Flor cultures (see Fig. S1 in the supplemental material).

Oxygen, total sulfide, and hydrogen fluxes were calculated according to Fick's first law of diffusion (*J* = –*D* ∂c/∂x). The diffusion coefficient (*D*) was 1.52 × 10^–9^ m^2^ s^–1^ for sulfide (29), 2.06 × 10^–9^ m^2^ s^–1^ for oxygen (29), and 3.67 × 10^–9^ m^2^ s^–1^ for hydrogen (30). The flux of a compound into the mat was equal to its consumption rate when the compound was depleted within the mat. When a compound diffused through the mat, its consumption rate was calculated as the absolute difference in the fluxes above and below the mat. Volumetric rates for hydrogen oxidation were calculated by assuming a constant mat thickness of 0.5 to 0.6 mm during the first 3 weeks. This thickness was estimated from images of 7-day-old cultures, which were the cultures in which the filaments can be seen best due to the high sulfur globule content (see Fig. 3A).

### Protein determination.

Total cell protein was measured as a substitute for Beggiatoa biomass as described previously (24, 31–33). The semiliquid top agar of a culture was sampled by pouring the entire volume into a 50-ml polypropylene tube. Residual agar that adhered to the walls of the culture tube was transferred by rinsing with 10 ml sterile artificial seawater. Centrifugation in a swing-out rotor at 5,000 × *g* (20 min) yielded a dense agar pellet of about 8 ml, in which the entire biomass was concentrated. The density of accompanying Pseudovibrio sp. FO-BEG1 cells in the thoroughly vortex-mixed pellet was determined in triplicate 10-μl subsamples using a Neubauer counting chamber. The remaining agar was hydrolyzed, and the protein was precipitated through incubation in 10% (wt/vol) trichloroacetic acid for 20 min at 90°C (24) followed by cooling at 4°C overnight. Four 2-ml subsamples were taken from each sample and centrifuged at 20,817 × *g* (10 min, 4°C). The supernatant was removed, and each pellet was dissolved and incubated in 0.7 ml 0.1 M NaOH (20 min, 55°C) to measure the protein content. The colorimetric protein assay (34) contained 0.5 ml sample or standard in 0.1 M NaOH, 0.5 ml 0.15 M HCl, and 0.35 ml dye reagent concentrate (Bio-Rad Laboratories, Inc., Hercules, CA, USA). Bovine serum albumin (2 to 10 μg ml^−1^) served as a standard. All measured protein concentrations were corrected for blanks (extractions from sterile top agar) and the contribution of Pseudovibrio sp. FO-BEG1 protein, considering the respective Pseudovibrio cell densities. The average protein content of Pseudovibrio sp. FO-BEG1 cells (290 ± 70 fg protein per cell) was determined separately. For this purpose, known amounts of axenically cultivated and washed Pseudovibrio sp. FO-BEG1 cells were added to a sterile mix of top agar and artificial seawater. The protein was extracted and quantified as described above.

### Estimation of sulfur inclusion density in filaments.

The density of sulfur inclusions was estimated semiquantitatively in Beggiatoa sp. 35Flor filaments that were grown in hydrogen-supplemented cultures with a low sulfide flux. Bacterial sulfur inclusions are highly light refractive, so the sulfur inclusion density correlates with the opaqueness of the filaments. At each time point, 333 to 385 filaments from three parallel cultures (105 to 134 filaments per culture) were inspected microscopically and assigned to one of five predefined categories of sulfur inclusion density (see Fig. 3E).

### Photography.

Photographs of culture tubes were taken with a Sony XCD-X710 digital camera (Sony, Tokyo, Japan), controlled by the image acquisition software IC Capture (The Imaging Source Europe GmbH, Bremen, Germany). Due to better visibility in print, negatives are shown. The brightness of all negatives was adjusted using the same modifications for all images contained in a figure. Different adjustments were used for different figures in order to achieve a good contrast when profiles were plotted on top of the photographs. Single filaments were photographed with a camera attached to a microscope (Sterni 2000-C, Zeiss, Germany), which was operated in bright-field mode.

## RESULTS

### Influence of molecular hydrogen on the migration behavior of Beggiatoa sp. 35Flor in gradient cultures with a low sulfide flux.

**FIG 2:**
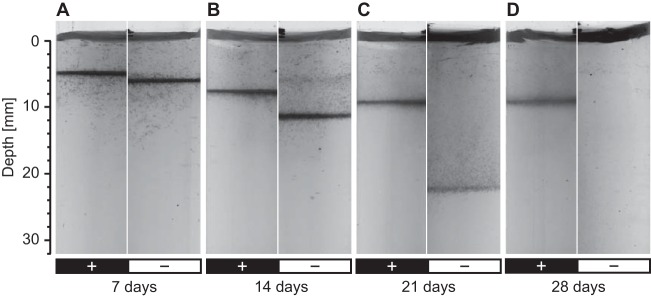
Position and appearance of Beggiatoa sp. 35Flor mats in hydrogen-supplemented and -unsupplemented oxygen-sulfide gradient media. The cultures were grown under low-sulfide-flux conditions over 4 weeks. Culture tubes were photographed after 7, 14, 21, and 28 days of growth in the presence (left panels; +) and absence (right panels; –) of a diffusional hydrogen gradient. The scale bar on the left indicates the depth below the air-agar interface.

Beggiatoa sp. 35Flor filaments grew in dense, opaque mats at the transition from oxic to sulfidic conditions when cultivated in agar-stabilized gradient media (Fig. 2). Irrespective of the presence or absence of hydrogen, these mats migrated downward in course of a 4-week incubation period in response to the progressive depletion of the bottom sulfide reservoir. However, the downward migration was considerably less pronounced in the presence of a diffusional hydrogen gradient (Fig. 2). While mats in hydrogen-supplemented cultures had not left the upper third of the top agar even after 4 weeks of growth, mats in hydrogen-unsupplemented cultures had already reached the bottom agar layer (Fig. 2D). No mat formation or growth of Beggiatoa sp. 35Flor could be observed in fresh sulfide-free gradient media supplemented with only oxygen and hydrogen.

### Hydrogen oxidation at the oxygen-sulfide interface in cultures with a low sulfide flux.

**FIG 3:**
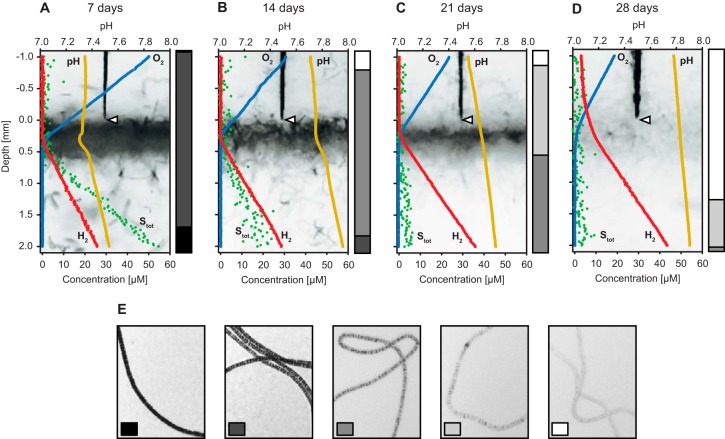
Development of chemical gradients and sulfur inclusion density in hydrogen-supplemented Beggiatoa sp. 35Flor cultures over 4 weeks. The cultures were grown in the presence of a low sulfide flux. (A to D) Microsensor profiles were recorded 7, 14, 21, and 28 days after inoculation. Profiles of oxygen (blue), hydrogen (red), pH (orange), and total sulfide (S_tot_; green) were determined at a vertical resolution of 20 μm. Photographs of the profiled mat sections are shown in the background. For each set of profiles, the tip of the microsensor (triangle) indicates the zero position at the mat surface to which all sensors were aligned. The bar graph next to each set of profiles shows a semiquantitative estimation of the relative sulfur inclusion density in Beggiatoa filaments at that point of time. This estimation considered five degrees of sulfur inclusion density, examples of which are shown in panel E. The standard deviation of sulfur inclusion density estimates in triplicate cultures was consistently below 10%. The density of sulfur inclusions decreased over time, resulting in an increasingly transparent appearance of the Beggiatoa mat.

Oxygen, sulfide, and hydrogen that diffused into Beggiatoa sp. 35Flor mats were consumed completely during the first 3 weeks of incubation (Fig. 3A to C). After 4 weeks, hydrogen was still oxidized, but the consumption was not complete and some hydrogen diffused through the mat (Fig. 3D). The zones of hydrogen and oxygen consumption overlapped at all times, and microsensor profiles showed no evidence of hydrogen oxidation in the anoxic section of the mat. The density of sulfur inclusions in Beggiatoa sp. 35Flor filaments decreased over the course of the incubation (Fig. 3A to D). After 4 weeks, about 75% of the filaments were devoid of visible sulfur inclusions and the remainder contained only a low level (Fig. 3D). The presence of ammonium at a concentration previously shown to inhibit nitrogen fixation in Beggiatoa sp. 35Flor (200 μM in the top agar; Henze, unpublished) did not affect hydrogen consumption in mats at the oxygen-sulfide interface (see Fig. S1 in the supplemental material). Hydrogen was not oxidized in axenic gradient cultures of Pseudovibrio sp. FO-BEG1, whereas it was consumed efficiently in parallel cocultures of Beggiatoa sp. 35Flor and Pseudovibrio sp. FO-BEG1 (see Fig. S2B), in which the average Pseudovibrio cell density was only 13% higher (*P* = 0.12; see Fig. S2A).

### Consumption rates of oxygen, sulfide, and hydrogen at the oxygen-sulfide interface.

**FIG 4:**
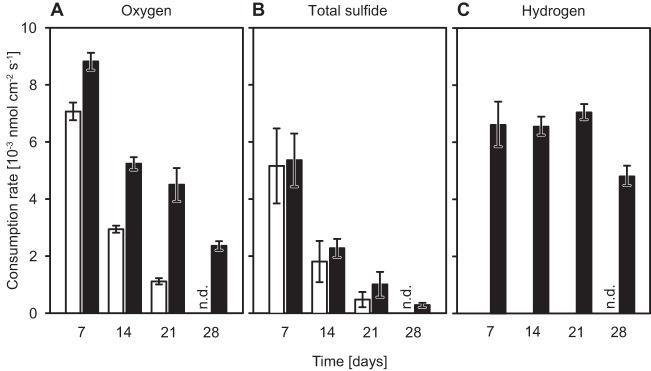
Average consumption rates of oxygen, total sulfide, and hydrogen in Beggiatoa sp. 35Flor mats over 4 weeks. Average consumption rates were determined weekly for hydrogen-supplemented (black bars) and -unsupplemented (white bars) cultures that were grown in the presence of a low sulfide flux. Rates of consumption of oxygen (A), total sulfide (B), and hydrogen (C) were calculated from profiles that were measured with a vertical resolution of 100 to 250 μm and that covered a distance of ca. 11 mm around the mat. The values for days 7, 14, and 21 are averages of consumption rates (± standard deviation) measured in six replicate cultures of two independent cultivations; values for day 28 are averages of consumption rates (± standard deviation) measured in triplicate cultures. Mats were absent from 28-day-old hydrogen-unsupplemented cultures, so consumption rates could not be determined (n.d.).

Average consumption rates of oxygen, total sulfide (S_tot_ = H_2_S + HS^−^ + S^2−^), and hydrogen were determined in hydrogen-supplemented and -unsupplemented Beggiatoa sp. 35Flor cultures over 4 weeks of incubation (Fig. 4). In both types of cultures, the consumption rates of total sulfide and oxygen decreased with incubation time due to the progressive depletion of the sulfide reservoir and the resulting downward migration of the mat. The average consumption rates of total sulfide were similar in the two types of cultures at all times (Fig. 4B). In contrast, the average oxygen consumption rate was always significantly higher (*P* ≤ 1.5 × 10^−5^) and decreased less over time in hydrogen-supplemented cultures (Fig. 4A). Measurements conducted within the first 3 weeks suggested a leveling off at a consumption rate of about 3 × 10^−3^ to 4 × 10^−3^ nmol O_2_ cm^−2^ s^−1^ in hydrogen-supplemented cultures, but a pronounced drop to circa 2.5 × 10^−3^ nmol O_2_ cm^−2^ s^−1^ occurred between weeks 3 and 4. The average hydrogen consumption rate in hydrogen-supplemented cultures remained fairly constant within the first 3 weeks but dropped markedly by week 4 (Fig. 4C), corresponding to the diffusion of hydrogen through the mat (Fig. 3D). A diffusion of hydrogen through the mat was always observed after about 4 weeks, but the fractions of hydrogen that passed through the mat at 28 days differed between independent cultivations.

### Influence of hydrogen oxidation on growth at the oxygen-sulfide interface.

**FIG 5:**
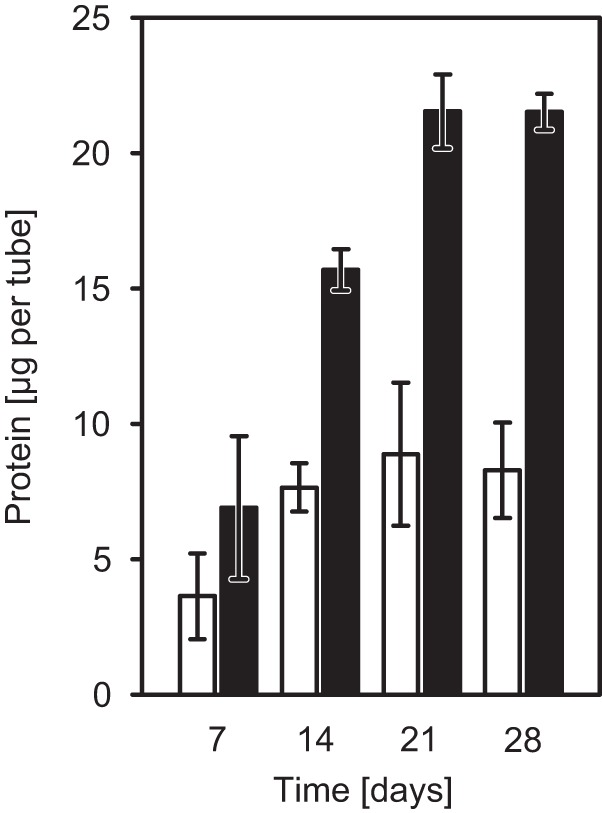
Influence of hydrogen oxidation on the growth of Beggiatoa sp. 35Flor. Beggiatoa sp. 35Flor protein levels were measured as a substitute for filament biomass in hydrogen-supplemented (black bars) and -unsupplemented (white bars) oxygen-sulfide gradient cultures. The cultures were grown in the presence of a low sulfide flux. The top agar from triplicate cultures was sampled weekly to determine the total protein content and the density of the accompanying Pseudovibrio sp. FO-BEG1 cells. Measured total protein amounts were subsequently corrected for the contribution of Pseudovibrio sp. FO-BEG1 protein, which accounted for 20% to 50% of the measured values (see Fig. S3 in the supplemental material).

Hydrogen-supplemented cultures grew faster and contained at least double the amount of Beggiatoa protein present in hydrogen-unsupplemented cultures at all time points (Fig. 5). In addition, hydrogen-supplemented cultures maintained growth for about 3 weeks, while hydrogen-unsupplemented cultures had already stopped growing after 2 weeks.

### Hydrogen oxidation under anoxic conditions in cultures with a high sulfide flux.

**FIG 6:**
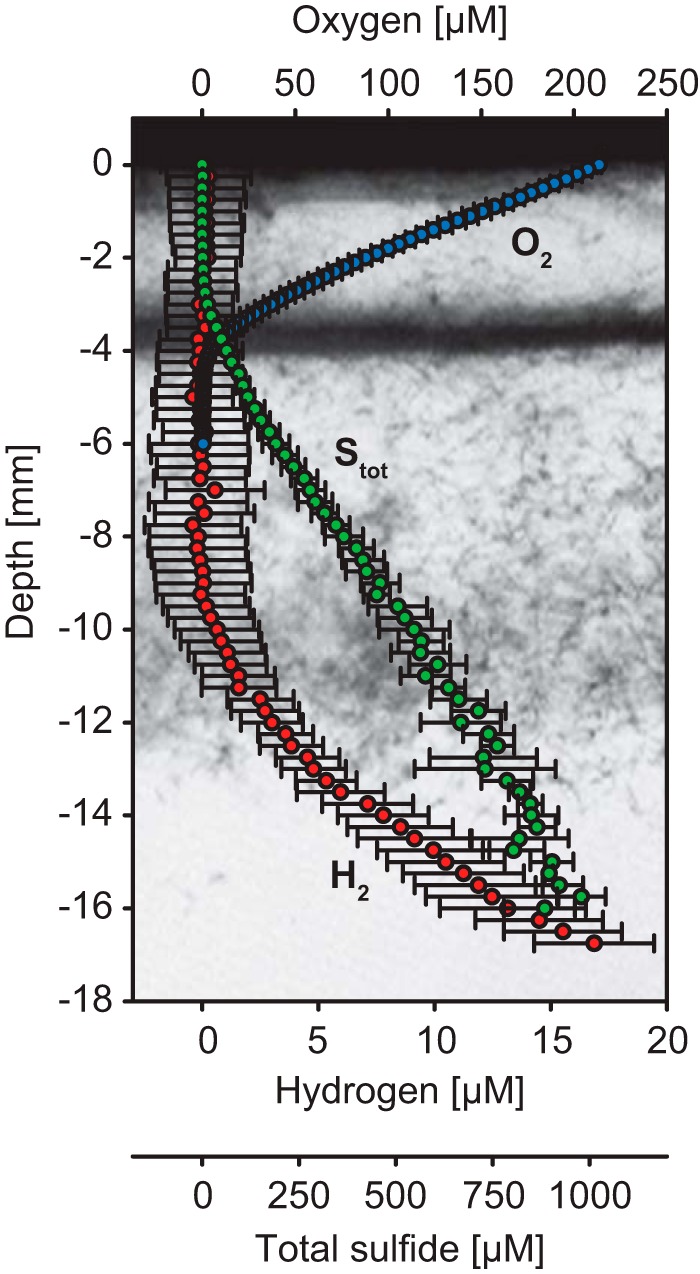
Hydrogen consumption under anoxic conditions in Beggiatoa sp. 35Flor cultures with a high sulfide flux. Oxygen (blue), hydrogen (red), and total sulfide (S_tot_; green) profiles were measured in hydrogen-supplemented cultures with vertical resolutions of 100 μm (O_2_) and 250 μm (H_2_, S_tot_). The plotted values are averages (± standard deviation) of measurements performed in three parallel cultures. The photograph in the background shows the filament distribution in a representative culture after 9 days of incubation when the profiles were measured. Depth values represent depth below the air-ager interface.

When Beggiatoa sp. 35Flor was grown in gradient cultures with a high sulfide flux, a subpopulation of filaments migrated from the oxygen-sulfide interface down into the anoxic section of the medium after about 1 week of incubation. Irrespective of the presence or absence of hydrogen, these filaments aggregated loosely in an anoxic horizon about 4 to 8 mm below the mat at the oxygen-sulfide interface. Microsensor profiles showed that added hydrogen was consumed within this horizon (Fig. 6). The profiles of H_2_S, pH, and total sulfide from hydrogen-supplemented cultures did not differ significantly from those of hydrogen-unsupplemented cultures, even though average H_2_S and total sulfide concentrations were minimally higher in the region of the anoxic subpopulation in hydrogen-supplemented cultures (see Fig. S4 in the supplemental material).

## DISCUSSION

We showed that a chemolithoautotrophic strain of the family Beggiatoaceae, Beggiatoa sp. 35Flor, consumed molecular hydrogen at the oxygen-sulfide interface. Microsensor profiles and rate measurements suggested that the strain oxidized hydrogen aerobically. With 5 to 17 nmol H_2_ per μg protein and hour or 417 to 523 nmol H_2_ per cm^3^ mat volume and hour (7 to 21 days; see Fig. S5 in the supplemental material), the average hydrogen oxidation rates were substantial and in fact exceeded the sulfide oxidation rates at all times (Fig. 4).

Hydrogen is a valuable electron donor for Beggiatoa sp. 35Flor, as illustrated by the significantly higher protein content in hydrogen-supplemented cultures (Fig. 5). Similarly to other members of the family Beggiatoaceae (24, 35), Beggiatoa sp. 35Flor is capable of nitrogen fixation (Henze, unpublished). Because this process releases hydrogen as a byproduct (28), many diazotrophs couple the expression of nitrogenase to the expression of uptake hydrogenases on a transcriptional level (36–39). Hydrogen oxidation occurring under conditions of repression of nitrogen fixation (see Fig. S1 in the supplemental material) showed, however, that Beggiatoa sp. 35Flor does not merely recycle internally produced hydrogen but is able to use externally supplied hydrogen as a genuine electron donor.

Beggiatoa sp. 35Flor grew in a defined coculture with Pseudovibrio sp. FO-BEG1, but several lines of evidence suggest that the Pseudovibrio strain did not contribute to the consumption of hydrogen. We did not observe hydrogen oxidation in gradient cultures that contained only Pseudovibrio sp. FO-BEG1 (see Fig. S2 in the supplemental material), and hydrogen oxidation was never observed in liquid cultures of the strain, irrespective of the incubation conditions tested (V. Bondarev, unpublished data). In addition, hydrogenase genes could not be identified in the essentially closed genome of Pseudovibrio sp. FO-BEG1 (Bondarev, unpublished).

Hydrogen oxidation clearly influenced the mat position, oxygen consumption, and growth of Beggiatoa sp. 35Flor. This is of particular importance for environmental studies, because it illustrates that the measurement of oxygen and sulfide gradients alone does not necessarily suffice to gain a comprehensive picture of Beggiatoaceae metabolism. In contrast, the use of alternative electron donors such as hydrogen or electron acceptors such as nitrate (40–44) can significantly influence biogeochemical gradients as well as the position of Beggiatoaceae populations with respect to these.

### Hydrogen versus sulfur as an electron donor at the oxygen-sulfide interface.

**FIG 7:**
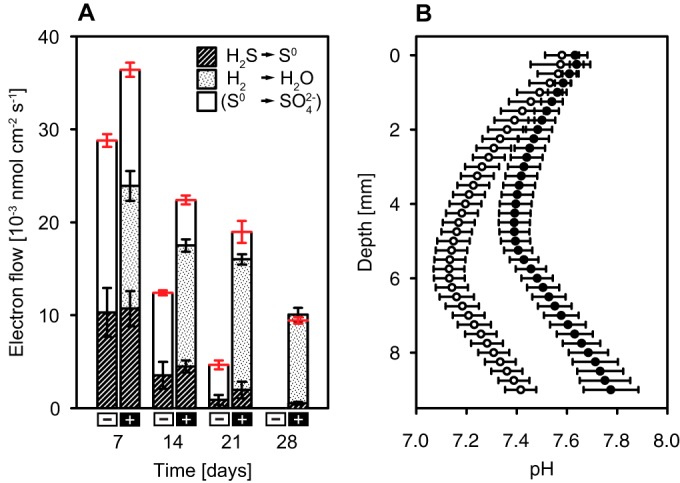
Influence of hydrogen oxidation on electron turnover and pH in Beggiatoa sp. 35Flor cultures. (A) Electron budgets in hydrogen-supplemented (+) and -unsupplemented (–) cultures over 4 weeks. The total electron demand (shown in red) was calculated based on the measured oxygen consumption rate and the estimated rate of CO_2_ fixation into biomass (<CH_2_O>). Weekly averages of CO_2_ fixation rates were estimated on the basis of the increase in Beggiatoa protein levels and a cell carbon-to-protein ratio of 1.13 (wt/wt; determined for closely related strain Beggiatoa sp. MS-81-6 under similar growth conditions; [31]). According to this estimation, CO_2_ fixation accounted for ≤6.4% of the total electron demand at all times. Hatched areas indicate the contribution of sulfide oxidation (2 electrons per H_2_S → S^0^) to the electron supply; dotted areas represent the contribution of hydrogen oxidation (2 electrons per H_2_). The electron demand, which cannot be fulfilled by the reactions described above, is most likely met by the oxidation of sulfur to sulfate (6 electrons per S^0^). (B) Average pH profiles (± standard deviation; *n* = 6) measured in hydrogen-supplemented (black) and -unsupplemented (white) cultures after 7 days of incubation. Mats in hydrogen-supplemented cultures were situated 5.2 to 6.2 mm below the air-agar interface; mats in hydrogen-unsupplemented cultures were located at a depth of 6.6 to 7.5 mm.

In order to assess the influence of hydrogen oxidation on the electron turnover in Beggiatoa sp. 35Flor, electron budgets were calculated on the basis of the measured consumption rates of oxygen, sulfide, and hydrogen as well as the estimated CO_2_ fixation rates (Fig. 7A). In hydrogen-unsupplemented cultures, the average contribution of sulfide oxidation to the total electron supply decreased from 36% to 20% within the first 3 weeks of incubation. The absolute rates of sulfide oxidation were similar in hydrogen-supplemented cultures, but the relative contribution to the total electron supply was lower because hydrogen-supplemented cultures showed an overall higher electron demand. Within the first 3 weeks, the average contribution of sulfide oxidation decreased from 30% to 11% of the total electron supply in hydrogen-supplemented cultures and dropped to only 6% after 4 weeks. Concurrently, the average contribution of hydrogen oxidation to the total electron supply increased from 36% after 1 week to 102% after 4 weeks. Hydrogen oxidation was already the main electron-supplying reaction after 2 weeks, fulfilling on average 59% of the total electron demand. Other electron donors and CO_2_ fixation as an electron sink were insignificant after 4 weeks such that hydrogen oxidation explained the total oxygen demand according to the Knallgas reaction (2H_2_ + O_2_ → 2H_2_O).

The total electron demand in hydrogen-supplemented and -unsupplemented cultures during the first 3 weeks was higher than what could be supplied by the oxidation of sulfide and hydrogen alone (Fig. 7A). This excess demand was most likely fulfilled by the oxidation of elemental sulfur to sulfuric acid as sulfur inclusions disappeared over time (Fig. 3), and pH profiles showed a pronounced acidification in the region of the mat (Fig. 7B). Sulfur oxidation in oxygen-sulfide gradient cultures of Beggiatoa sp. 35Flor was recently also demonstrated by the production of large amounts of sulfate (45). Notably, the excess electron demand was lower in hydrogen-supplemented cultures throughout the incubation (Fig. 7A). Together with a less pronounced acidification of the medium (Fig. 7B), this suggests that less sulfur was oxidized to sulfuric acid in the presence of hydrogen. In addition to the production of sulfuric acid, higher CO_2_ fixation rates (Fig. 5) contribute to higher pH values in hydrogen-supplemented cultures. However, it is unlikely that the observed pH difference resulted mainly from differences in CO_2_ fixation rates, because the estimated contribution of CO_2_ fixation to the total electron demand was low in general (≤6.4%; Fig. 7).

Overall, the influence of hydrogen oxidation on the sulfur metabolism of Beggiatoa sp. 35Flor points to a very efficient and purposeful use of the different electron donors in an environment, in which sulfide toxicity, competition for resources, and fluctuating supplies with oxidants and reductants are the major challenges. Sulfide and hydrogen, which cannot be stored, are oxidized immediately when available, while sulfur may be kept in reserve when the current energy requirements can be met by using other electron donors.

### Aerobic hydrogen oxidation occurs in the presence of reduced sulfur compounds.

The presented results clearly show that Beggiatoa sp. 35Flor used energy from aerobic hydrogen oxidation for growth when reduced sulfur compounds were available. In contrast, growth on hydrogen in the absence of reduced sulfur compounds could not be shown. The apparent inability of hydrogen to support growth as an exclusive electron donor was unexpected, given that electrons from hydrogen (46, 47) enter the electron transport chain either on the same level as or upstream of electrons from reduced sulfur compounds (48–50) and thus should be able to support at least the same metabolic processes. So far, the reason for absent or discontinued growth of Beggiatoa sp. 35Flor on hydrogen and oxygen alone is unclear. Possible explanations are the potential inability to assimilate sulfate, the accumulation of waste products in older cultures, or the missing abiotic oxygen removal by sulfide and the resulting lack of a microoxic niche in fresh sulfide-free gradient media.

### *Beggiatoa* sp. 35Flor oxidizes hydrogen also under anoxic conditions, presumably through sulfur respiration.

Beggiatoa sp. 35Flor filaments did not oxidize hydrogen only aerobically in mats at the oxygen-sulfide interface (Fig. 3). In cultures with a high sulfide flux, hydrogen was also oxidized in the fully anoxic section of the medium by a subpopulation of filaments that had migrated downward from the oxygen-sulfide interface (Fig. 6). Several members of the family Beggiatoaceae are known to store nitrate in large amounts and use it as an alternative electron acceptor under anoxic conditions (e.g., 40, 43, 44, 51). However, nitrate can be excluded as an electron acceptor in the present study. Gradient media for precultures and experiments were prepared without fixed nitrogen compounds, and Beggiatoa sp. 35Flor filaments from such precultures were previously shown to be free of NO_X_ compounds (25). Hence, neither external nor internal nitrate was available for hydrogen oxidation under anoxic conditions.

In addition to the use of nitrate, several members of the family Beggiatoaceae are known to use stored sulfur as an electron acceptor under short-term anoxic conditions. Previous studies showed that sulfur respiration in Beggiatoaceae can be supported by organic electron donors such as acetate (52) and internally stored polyhydroxyalkanoates (7, 25) but also by molecular hydrogen (7). Sulfur respiration was recently shown in the strain Beggiatoa sp. 35Flor by Schwedt et al. (25) under incubation conditions very similar to the ones used here. Schwedt and colleagues showed that Beggiatoa sp. 35Flor filaments that had migrated into the anoxic section of a gradient medium under high sulfide fluxes reduced stored sulfur with stored polyhydroxyalkanoates. We assume that the same population of filaments as was studied by Schwedt et al. (25) used sulfur also as an electron acceptor for hydrogen oxidation under anoxic conditions in our experiments. Total sulfide profiles recorded in hydrogen-supplemented and -unsupplemented cultures did, however, not show significantly higher sulfide concentrations in the region of the anoxic subpopulation when hydrogen was present (see Fig. S3 in the supplemental material). This may have been due to the fact that the increase in sulfide production through hydrogen oxidation was too low compared to the background sulfide flux and the variability among replicate cultures. The average expected sulfide production rate, which is equal to the average measured hydrogen consumption rate (H_2_ + S^0^ → H_2_S; 1.16 × 10^−3^ nmol cm^−2^ s^−1^), was only 11% of the background sulfide flux (10.50 × 10^−3^ nmol cm^−2^ s^−1^) in hydrogen-supplemented cultures. The standard deviation of total sulfide concentrations was 7% to 18% (*n* = 3; hydrogen-supplemented cultures) and 9% to 23% (*n* = 3; hydrogen-unsupplemented cultures) of the average values in the region of the anoxic subpopulation located at a depth of 8 to 12 mm. For this reason, a significant increase in sulfide production through hydrogen oxidation may not have been detectable.

Sulfur respiration in Beggiatoaceae has been suggested to serve two purposes: the generation of metabolic energy under short-term anoxic conditions (7, 52) and the disposal of excess internal sulfur to avoid cell rupture (25). The use of hydrogen as an electron donor would enable an uncoupling of sulfur respiration from the oxidation of organic carbon compounds, thus leading to a higher flexibility in energy generation and sulfur disposal under anoxic conditions.

Thus, hydrogen oxidation has the potential of increasing the ecophysiological plasticity of Beggiatoa sp. 35Flor and possibly of other members of the family Beggiatoaceae in two ways, both of which are tightly coupled to the sulfur metabolism. In the presence of a low sulfide flux and electron acceptors with a more positive redox potential such as oxygen, hydrogen can partially replace sulfur as an electron donor and thereby increase the amount of sulfur available for storage. In contrast, hydrogen may support sulfur respiration and disposal under conditions of high sulfide flux and anoxia in order to provide metabolic energy and prevent physical damage from excessive sulfur accumulation.

### Environmental relevance of hydrogen oxidation for members of the family Beggiatoaceae.

A variety of biotic and abiotic environmental processes are associated with the production of molecular hydrogen (19). Nevertheless, significant amounts of hydrogen are probably available to Beggiatoaceae in only certain environments. Members of this family are very often found in organic-rich sediments, in which hydrogen is produced by fermentation. However, the preferred habitat of Beggiatoaceae, the oxygen-sulfide interface, is usually well and permanently separated from the zone of hydrogen production in these sediments. Even though large quantities of hydrogen are produced by fermentative processes in deeper, anoxic layers, H_2_ is rapidly and efficiently reoxidized by the local community of hydrogenotrophic prokaryotes (20). Beggiatoaceae, which populate the oxygen-sulfide interface, are therefore unlikely to experience high concentrations or fluxes of hydrogen in such systems. In contrast, nitrate- or sulfur-respiring members of the family, which are residing in or traveling through fermenting sediment layers, could exploit hydrogen as an electron donor.

The hypersaline cyanobacterial mats of the Guerrero Negro evaporation lagoons (Baja California Sur, Mexico) are a prominent example of an environment in which large amounts of hydrogen are frequently available to members of the Beggiatoaceae. The biogeochemical conditions in these mats follow a strong diel cycle (53–56), which involves the presence of exceptionally high hydrogen concentrations at the mat surface during nighttime (57). Reacting to the changing biogeochemical conditions, filamentous Beggiatoaceae migrate to the anoxic and sulfidic surface of the Guerrero Negro mats at night (58, 59) and thus are regularly exposed to high hydrogen concentrations. Extensive cyanobacterial mats resembling those of the Guerrero Negro lagoons were present on earth for most of life's history, once dominating the biosphere (53, 60, 61). Substantial genetic exchange between cyanobacteria and Beggiatoaceae (62, 63) strikingly evidences a historically frequent co-occurrence of these taxa. This suggests that hydrogen transfer from nitrogen-fixing and fermenting cyanobacteria to members of the family Beggiatoaceae could indeed be an ancient and once-widespread process.

In addition, chemosynthetic ecosystems in the deep sea are sites at which hydrogen, specifically, H_2_ of geothermal origin, could potentially serve as a source of metabolic energy for Beggiatoaceae. Members of the family are regularly encountered in the deep sea at sites of hydrothermal fluid flow (e.g., 64–69), and hydrogen is extruded at several of such places (18, 70, 71). In fact, H_2_ of geothermal origin was suggested to be a key energy source in deep-seawater masses (17) and has been shown to fuel CO_2_ fixation in sulfide-oxidizing endosymbionts of deep sea mussels (18). Yet seep-dwelling populations of Beggiatoaceae have apparently never been tested for exposure to or even consumption of H_2_. Similarly to submarine sites of hydrothermal fluid flow, members of the Beggiatoaceae thrive in terrestrial sulfidic springs (1, 5, 72, 73), sites at which hydrogen is frequently emitted (74). However, further studies are necessary to evaluate the importance of molecular hydrogen for members of the family Beggiatoaceae on a broader scale. These studies will need to investigate the availability of H_2_ to environmental populations as well as the ability of different strains to oxidize this electron donor.

## Supplementary Material

Supplemental material
