# Supplementary material for: Oxidation of Molecular Hydrogen by a Chemolithoautotrophic Beggiatoa Strain
Source: Appl Environ Microbiol. 2016 Apr 4;82(8):2527–36. doi: 10.1128/AEM.03818-15 (PMC4959497; doi:10.1128/AEM.03818-15)
Supplement: Supplemental material [file supp_82_8_2527__index.html]

Oxidation of Molecular Hydrogen by a Chemolithoautotrophic Beggiatoa Strain — Supplemental material 

# Oxidation of Molecular Hydrogen by a Chemolithoautotrophic Beggiatoa Strain

## Supplemental material

- Supplemental file 1 -

  Influence of a fixed nitrogen source on hydrogen oxidation by *Beggiatoa* sp. 35Flor (Fig. S1); test for hydrogen oxidation by *Pseudovibrio* sp. FO-BEG1 (Fig. S2); *Beggiatoa* sp. 35Flor and *Pseudovibrio* sp. FO-BEG1 protein content in oxygen-sulfide gradient cultures over four weeks of incubation (Fig. S3); H2S, pH, and total sulfide profiles in *Beggiatoa* sp. 35Flor cultures with a high level of sulfide flux (Fig. S4); development of the hydrogen consumption rate normalized to *Beggiatoa* sp. 35Flor biomass or mat volume over four weeks of incubation in the presence of a low level of sulfide flux (Fig. S5).

  PDF, 464K
