# Supplementary material for: Oxidation of Molecular Hydrogen by a Chemolithoautotrophic Beggiatoa Strain
Source: Appl Environ Microbiol. 2016 Apr 4;82(8):2527–36. doi: 10.1128/AEM.03818-15 (PMC4959497; doi:10.1128/AEM.03818-15)
Supplement: Supplemental material [file AEM.03818-15_zam999117085so1.pdf]

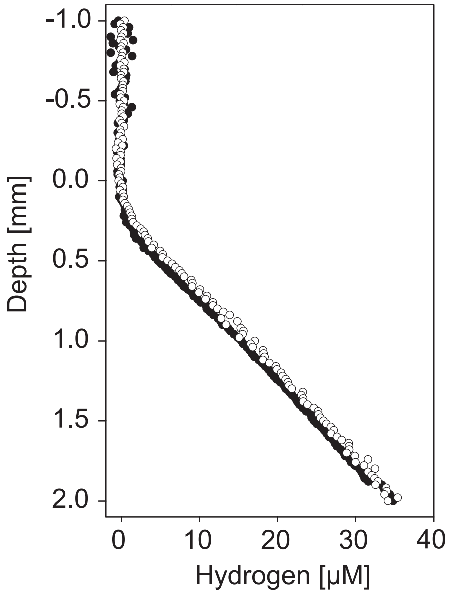

Figure S1 | Influence of a fixed nitrogen source on hydrogen oxidation by *Beggiatoa* sp. 35Flor. Oxygen-sulfide gradient media with a diffusional hydrogen gradient were prepared without the addition of fixed nitrogen compounds (○) or with an initial ammonium concentration of 200 μM in the top agar (●). This concentration was previously shown to inhibit nitrogen fixation in *Beggiatoa* sp. 35Flor cultures (A.-T. Henze, unpublished data). Hydrogen profiles were recorded after 7 days of incubation. The position zero indicates the mat surface.

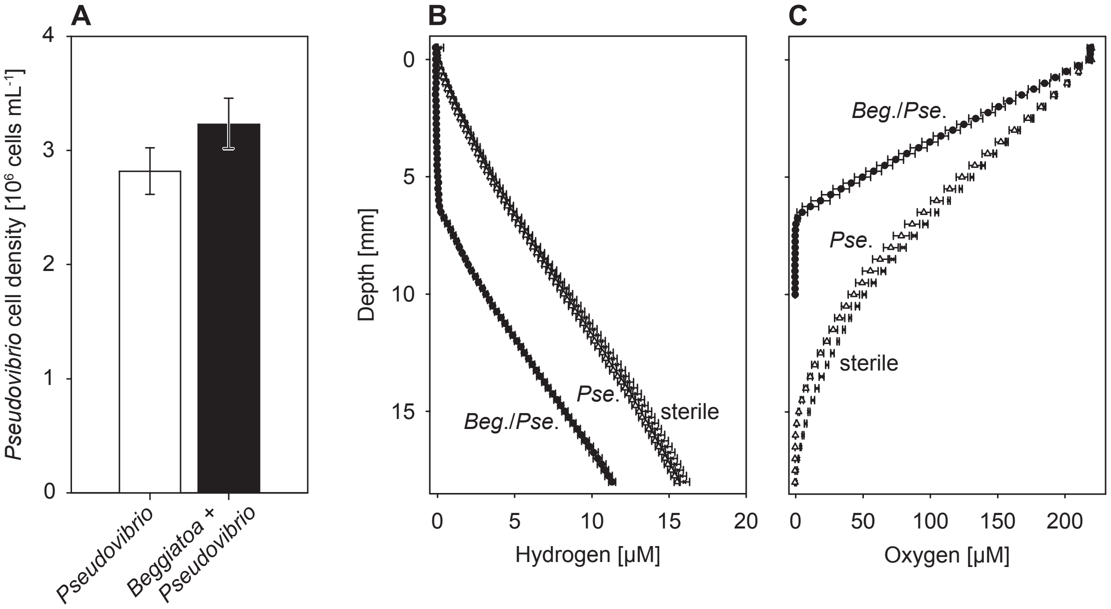

Figure S2 | Test for hydrogen oxidation by *Pseudovibrio* sp. FO-BEG1. Oxygen-sulfide gradient media with a low sulfide flux and a diffusional hydrogen gradient were inoculated with either a mixed *Beggiatoa* sp. 35Flor/ *Pseudovibrio* sp. FO-BEG1 suspension or an axenic *Pseudovibrio* sp. FO-BEG1 suspension of the same cell density. Sterile media were kept as controls and measurements were performed after eight days of growth. (**A**) *Pseudovibrio* cell densities are shown as averages (± standard deviation) of counts in three parallel cultures. The cell densities were normalized to the entire top agar but cells concentrate in *Beggiatoa*/ *Pseudovibrio* (black bar) and *Pseudovibrio* (white bar) cultures at the oxygen-sulfide interface (A. Fink, unpublished data). (**B+C**) Hydrogen and oxygen profiles measured in *Beggiatoa*/ *Pseudovibrio* (●), *Pseudovibrio* (△), and sterile cultures (×) are shown as averages (± standard deviation; $n = 4$). Hydrogen profiles were corrected for the sulfide-derived background recorded by the cross-reactive hydrogen sensor. Moderately higher oxygen consumption rates in *Pseudovibrio*-inoculated cultures relative to sterile media are evident. However, this effect was previously also observed in hydrogen-unsupplemented media and most likely results from the oxidation of reduced sulfur compounds or agar impurities by *Pseudovibrio* sp. FO-BEG1 (A. Fink, unpublished data). The y-axis gives the depth below the air-agar interface.

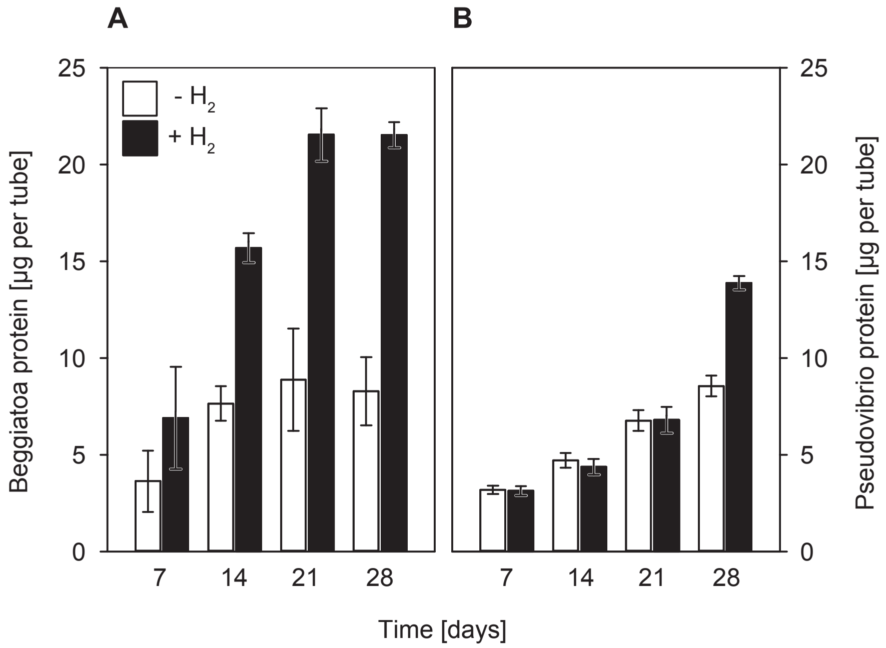

Figure S3 | *Beggiatoa* sp. 35Flor and *Pseudovibrio* sp. FO-BEG1 protein content in oxygen-sulfide gradient cultures over four weeks of incubation. Hydrogen-supplemented (black bars) and hydrogen-unsupplemented (white bars) cultures were grown in presence of a low sulfide flux. The amount of *Pseudovibrio* protein per culture tube (B) was calculated based on the determined *Pseudovibrio* cell density and the average protein content per *Pseudovibrio* cell. This amount was subtracted from the total measured protein to obtain the amount of *Beggiatoa* protein per culture tube (A).

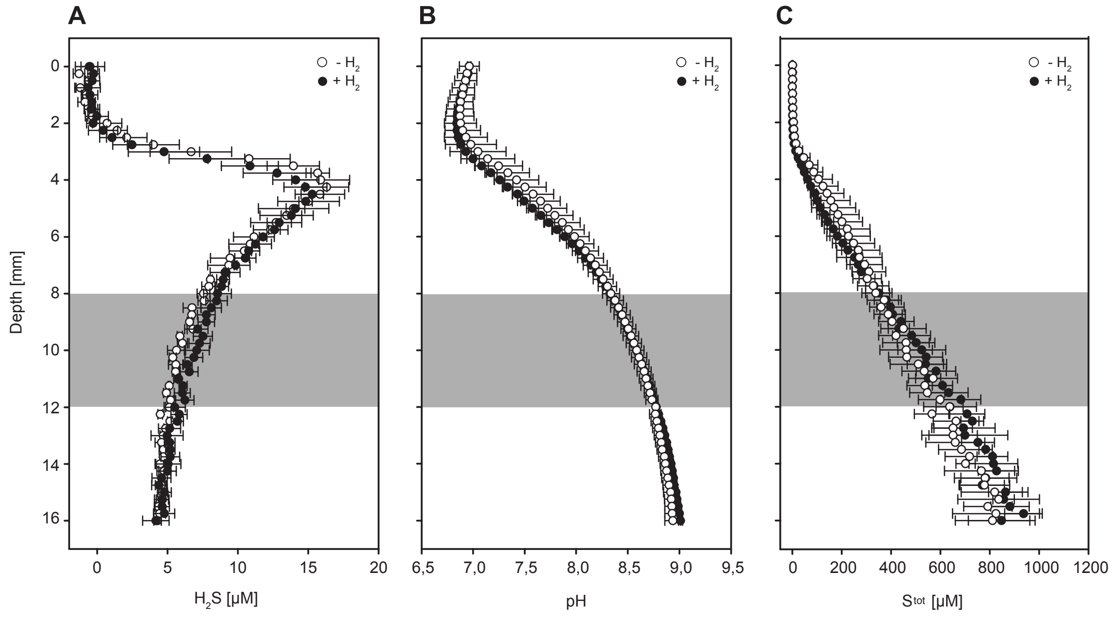

Figure S4 | H_2S, pH, and total sulfide profiles in *Beggiatoa* sp. 35Flor cultures with a high sulfide flux. H_2S **(A)**, pH **(B)**, and total sulfide **(C)** profiles were measured in hydrogen-supplemented (○) and -unsupplemented (●) *Beggiatoa* sp. 35Flor cultures after nine days of incubation. The plotted values are averages (± standard deviation) of measurements in three replicate cultures. The horizon, in which the anoxic subpopulation showed the highest filament density, is shaded in grey.

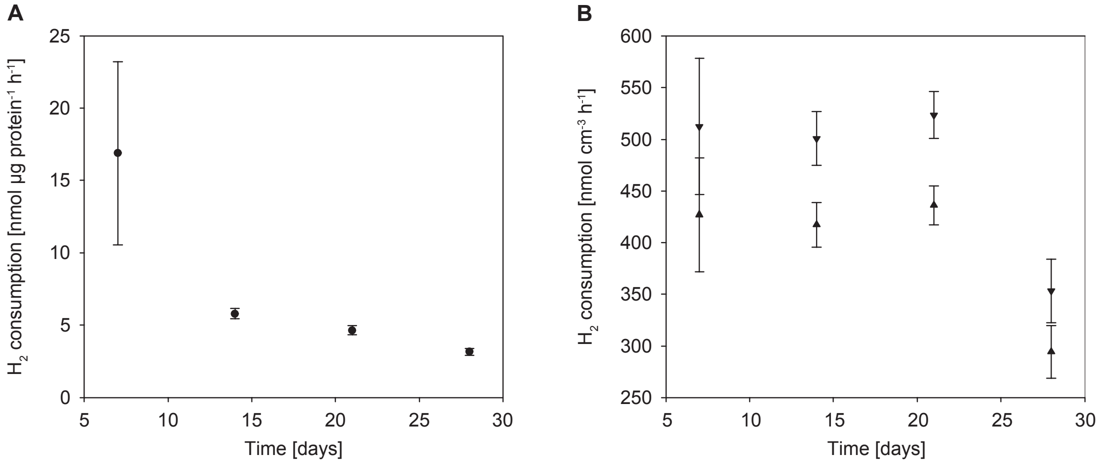

Figure S5 | Development of the hydrogen consumption rate normalized to *Beggiatoa* sp. 35Flor biomass or mat volume over four weeks of incubation in presence of a low sulfide flux. **(A)** Hydrogen consumption rate per μg *Beggiatoa* protein in mats at the oxygen-sulfide interface. **(B)** Hydrogen consumption rate per cubic centimeter mat volume for mat thicknesses of 0.5 mm (▼) and 0.6 mm (▲).
